# Social class, social mobility and alcohol-related disorders in Swedish men and women: A study of four generations

**DOI:** 10.1371/journal.pone.0191855

**Published:** 2018-02-14

**Authors:** Anna Sidorchuk, Anna Goodman, Ilona Koupil

**Affiliations:** 1 Department of Public Health Sciences, Karolinska Institutet, Stockholm, Sweden; 2 Department of Clinical Neurosciences, Karolinska Institutet, Stockholm, Sweden; 3 Department of Public Health Sciences, Stockholm Unviersity, Stockholm, Sweden; 4 Faculty of Epidemiology and Population Health, London School of Hygiene and Tropical Medicine, London, United Kingdom; Alcohol Research Group, UNITED STATES

## Abstract

**Objectives:**

To investigate whether and how social class and social mobility in grandparents and parents predict alcohol-related disorders (ARDs) in males and females aged 12+ years, and whether intergenerational social prediction of ARDs varies across time periods.

**Methods:**

The study sample included four successive generations (G) of Swedish families from the Uppsala Birth Cohort Multigenerational Study: G0 born 1851–1912; G1 born 1915–1929; G2 born 1940–1964 and G3 born 1965–1989. Two study populations were created, each consisting of grandparents, parents and offspring: population I ‘G0-G1-G2’ (offspring *n* = 18 430) and population II ‘G1-G2-G3’ (offspring *n* = 26 469). Registers and archives provided data on ancestors’ socio-demographic factors and ARD history, together with offspring ARD development between 1964–2008. Cox regression models examined the hazard of offspring ARD development according to grandparental social class and grandparental-to-parental social trajectories, controlling for offspring birth year, grandmother’s and mother’s marital status and parental ARDs.

**Results:**

Disadvantaged grandparental social class predicted increased ARD risk in offspring in population I, although the effect attenuated and became non-significant in males after adjusting for parental characteristics (adjusted hazard ratio (HR) = 1.80 (95%CI; 1.07, 3.03) in females, HR = 1.32 (95%CI; 0.93, 1.89) in males). In population II, no increase in ARD risk by grandparental social was evident. In both populations, males were at the highest ARD risk if both parents and grandparents belonged to disadvantaged social class (population I: HR = 1.82 (95%CI; 1.22–2.72); population II: HR = 1.68 (95%CI; 1.02–2.76)).

**Conclusions:**

Intergenerational social patterning of ARDs appears to be time-contextual and gender-specific. The role of grandparental social class in developing ARDs in grandchildren seems to decline over time, while persistent grandparental-to-parental social disadvantage remains associated with higher ARD risk in males. When targeting higher risk groups, continuity of familial social disadvantage, particularly among males, should be considered.

## Introduction

Despite substantial public health awareness and extensive policy enforcement [1], alcohol use is nonetheless currently ranked as the sixth leading risk factor for the burden of disease in high-income countries, and the ninth leading factor worldwide [2]. Although Sweden today has one of the lowest levels of alcohol consumption in Europe [1, 3], alcohol accounts for 2.6% of the total disease burden in the country (4.2% in men vs. 0.9% in women) with this mainly reflecting premature deaths [2]. With substantial social costs, increasing hospitalization for alcohol poisoning among young adults and rising alcohol-attributable mortality in people of advanced age [3], the prevention of unhealthy use and reduction of harm caused by alcohol are recognized as one of major strategic areas in the Swedish national public health policy [3, 4].

Understanding the origins of social inequalities and the mechanisms that can potentially compensate for the disparities in alcohol-related disorders (ARDs) is a key step in targeting and implementing public health interventions [5]. A social gradient in alcohol-related morbidity and mortality is well documented in single-generational studies, with an elevated risk of ARDs generally found to be related to unfavorable socioeconomic circumstances [6–9]. Substantial socio-economic differences in ARDs exist despite levels of alcohol *consumption* showing relatively minor differences between social economic groups, a contrast that has been labeled as the ‘alcohol harm paradox’. In the light of this paradox, it has been proposed that, social disadvantage *per se* is predictive of alcohol-related health consequences through the exposure to poor material and psychosocial resources, over and beyond the impact of drinking patterns [7, 9]. One mechanism for this increased vulnerability to developing ARDs may involve the accumulated influence of a number of factors operating in early life, including poverty-related health deficiencies, negative rearing conditions such as inefficient parenting and childhood household dysfunction, or lack of resources for optimal educational and social achievements [10–12].

A similar socio-economic gradient is observed across generations, with low parental social status associated with ARD development in subsequent generation in most studies [13–16], although inverse and non-significant associations are also reported [17, 18]. As social disadvantage appears to cluster in families across multiple generations [19], it is important to address the origins of inequalities in a broader perspective, beyond the parent-child relationship, to better understand the nature of inequalities and clarify the extent to which family social context predicts developing offspring’s ARDs. Such understanding may have implications for optimizing the design and delivery of public health initiatives given the demonstrated effectiveness of selective, personality-targeted prevention of alcohol misuse [20] as well as the differential effect of preventive interventions across social strata [21]. The latter further highlights a need to address intergenerational social mobility and to explore whether vulnerability to ARDs depends on not just current socio-economic position but also on the trajectory of social mobility.

Previous research on social mobility has focused on the comparative importance of childhood and adulthood socioeconomic circumstances, and has reported mixed results [14–16]. Swedish studies report grandparental education and income to be highly predictive of the corresponding facets in parents and grandchildren indicating intergenerational transfer of human capital [22, 23]. Additionally, sociological research documents direct grandparent-to-grandchild transmission of intangible resources (e.g. cultural capital) [24, 25] and studies on intergenerational continuity of substance use habits indicate that grandparents are role models regarding values, socializing practices and risk aversion [26–28]. Swedish and Australian studies have found grandparental social disadvantage to be significantly associated with grandchildren’s impaired cognitive and emotional development, independent of parental socioeconomic circumstances [29, 30]. These results are noteworthy in light of the evidence linking low cognitive ability and behavioural problems to elevated risk of ARDs [31–33]. In addition to these seemingly direct effects of grandparental social conditions, there are also likely to be indirect pathways mediated via the parental generation. For example, grandparents may transfer parenting practices and health-related behaviours to the parent generation, along with resources and capacities to buffer physical, psychological and financial stressors; and these parents may, in their turn, reproduce the strategies while bringing up their own children [34–36].

Encompassing data over the life courses of several generations additionally enables examination of whether the social patterning of ARDs varies across different historical periods. In Sweden, historical trends in alcohol policies, the national level of social inequality and attitudes to alcohol have contributed to changes in drinking patterns and alcohol-attributable deaths [37–40], and as such it is plausible that social inequalities in ARDs might vary over time.

To our knowledge there are no published studies that embrace three or more generations to investigate the social gradient in ARDs. Our study seeks to do this using data from the Uppsala Birth Cohort Multigenerational Study (UBCoS Multigen) [41], adopting a multigenerational and time contextual perspective. Established to explore life course and intergenerational patterning of social inequalities in health, and currently spanning five generations [42, 43], the UBCoS Multigen provides a unique opportunity of prospectively assessing ARD development in offspring in relation to prior generations’ social determinants measured in different periods of Swedish history. In our current analysis, we aimed to assess whether and how social class and social mobility in grandparents and parents predict ARDs in offspring, and to what extent the associations can be explained by other grandparental and parental socio-demographic factors as well as by the parents’ own ARD history. We also focused on investigating whether intergenerational social prediction of offspring’s ARDs varies across different time periods.

## Materials and methods

### Study population

The study cohort consisted of four successive generations of Swedish families from the UBCoS Multigen [41]. The cohort composition and data linkages have been described in detail elsewhere [42, 43]. Briefly, the original Uppsala Birth Cohort study sample comprised all 14 192 live births occurring in 1915–1929 in the Uppsala University Hospital, Sweden (henceforth “G1s” for “generation one”). This population was identified through archived obstetric records, and these records also contained information on the G1s’ parents (“G0s”), born 1851–1912. Among the 14 192 G1s, 13 865 (98%) were successfully traced through parish archives until death, emigration or until being assigned a unique personal number. Of these, 12 168 G1s were alive and living in Sweden in late 1940s, and so received unique personal numbers at that time. Through personal numbers all G1s were linked with their children (“G2s”) and grandchildren (”G3s”) using the Swedish Multigenerational register [44]. To complete family linkage, the G1s’ and G2s’ partners, i.e. the other biological or adoptive parents of their descendants, were traced in the same register [42, 43].

**Fig 1:**
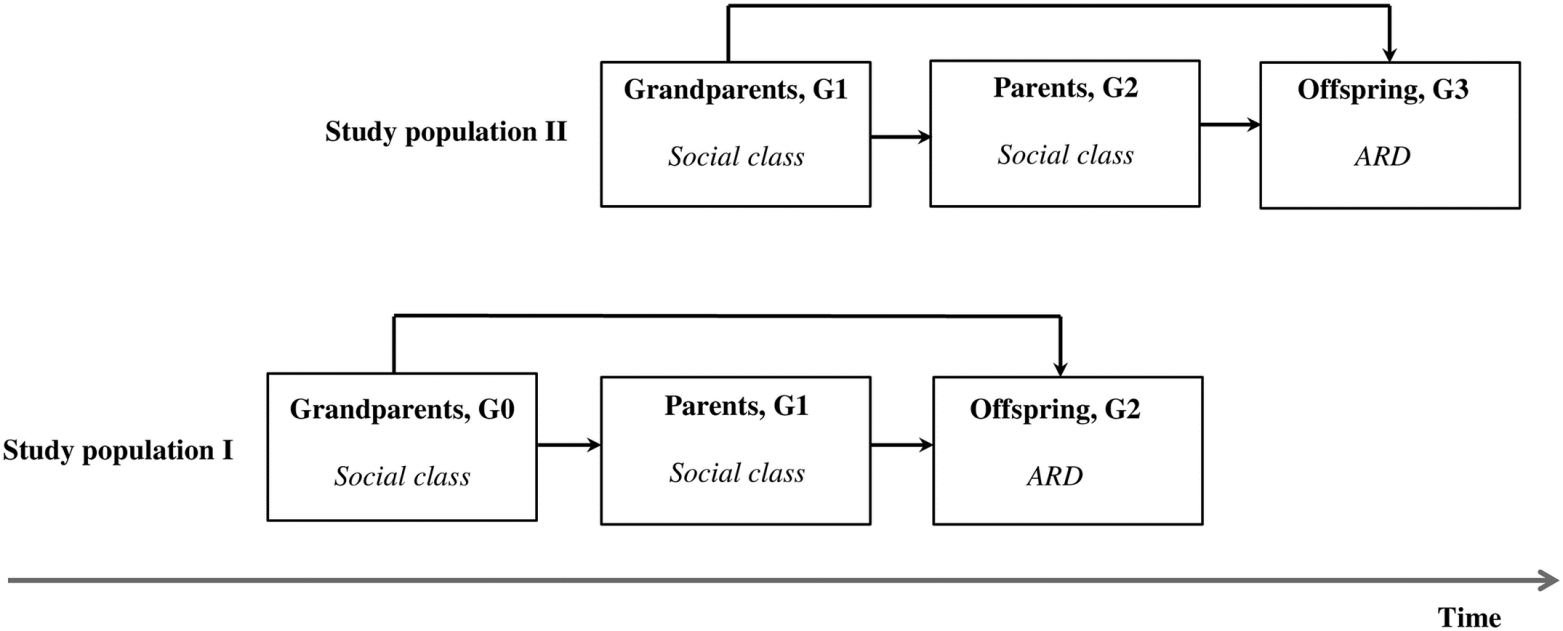
Intergenerational profile of study population I and population II: UBCoS Multigen. Abbreviations: ARD = alcohol-related disorders, G = generation, UBCoS Multigen = the Uppsala Birth Cohort Multigenerational Study. Boxes represent generations under analyses with established biological relations and arrows denote potential pathways between generations. Variables *in Italics* signify independent (social class) and dependent (ARD) variables measured in corresponding generations.

For the purpose of the present analysis, two study populations were created, each consisting of three successive generations to be analysed separately as the population I (G0-G1-G2) and population II (G1-G2-G3). The members of each population were respectively denoted as grandparents, parents and offspring. Fig 1 outlines the populations’ composition along with potential pathways of interest.

In population I, the starting point for the offspring generation was 19 251 G2 members born in 1940–1964. In population II, the starting point was 27 646 G3 individuals born 1965–1989. Of these, we excluded adopted offspring (n = 64 in population I, n = 134 in population II), those who died or emigrated before the start of follow-up (n = 78 and n = 597, respectively), or those with missing data on the study predictors or covariates (n = 679 and n = 446, respectively). This yielded an analytical sample of 18 430 G2s in population I (95.8% of those eligible) and 26 469 G3s in population II (95.7% of those eligible). Table 1 describes the populations’ profile and outlines the completeness of family lineages. Data on mothers were available for all offspring included in analytical sample, as were data on almost all fathers. By contrast, most individuals had data only on either the maternal or the paternal grandparents; the proportion with data on both lineages was only 9.1% in population I and 4.5% in population II. The study was approved by the Regional Ethical Review Board, Karolinska Institutet, Stockholm (Dnr 03–117, Dnr 04-944T, Dnr 2009/1115-32, Dnr 2009/1830-32, and Dnr 2014/2058-31/5). Prior to the analysis all data were fully anonymised and de-identified.

**Table 1:** Cohort profile within the population I and II: The Uppsala Birth Cohort Multigenerational Study (UBCoS Multigen).

Characteristics	Population I offspring (G2)			Population II offspring (G3)		
	n	% of initial sample	% of analytical sample	n	% of initial sample	% of analytical sample
**Offspring initial sample**						
Years of birth	1940–64			1965–89		
Total number	19 251	100		27 646	100	
Excluded from the analysis	821	4.2		1177	4.3	
*Reasons for exclusion*:						
Adopted	64	0.3		134	0.5	
Died or emigrated before the start of follow-up (January 1, 1964) or before the age of 12	78	0.4		597	2.2	
Missing predictors or covariates^a^	679	3.5		446	1.6	
**Offspring analytical sample**						
Total number	18 430	95.8	100	26 469	95.7	100
Males	9420		51.1	13 575		51.3
Females	9010		48.9	12 894		48.7
**Offspring with identified parents**^b^ **/grandparents**^c^						
Mothers	18 430		100	26 469		100
Fathers	18 065		98.0	26 348		99.5
Paternal grandparents only	8394		45.5	13 097		49.5
Maternal grandparents only	8365		45.4	12 181		46.0
Maternal and paternal grandparents	1671		9.1	1191		4.5

^a^ Excluded if missing data on any of the following variables: grandparental and parental social class, grandmother’s and mother’s marital status, parental education, parental income, father’s and mother’s alcohol-related disorders.

^b^ Parents: population I–G1; population II–G2.

^c^ Grandparents: population I–G0; population II–G1.

### Predictors

Grandparental and parental social class variables were constructed based on individual occupation using the Swedish socioeconomic classification (SEI) [45]. Information was retrieved from archived data, primarily hospital obstetric records, for the G0s and from the Population and Housing Census 1960 and 1990 for the G1s and G2s, respectively. To increase comparability in the measures of social classes across all generations, and to limit the number of grandparental-to-parental social trajectories, occupational social classes were categorised as “highly advantaged”, “advantaged” and “disadvantaged”. Details on categorization are presented in S1 and S2 Tables footnotes. If data were available on both maternal and paternal occupations, the highest social class within a couple was used to create a single measure of parental social class for each offspring. In most cases, data on grandparents were available for only one lineage, and the variable “highest grandparental social class” was created using the available lineage. If both lineages were identified, one lineage was selected at random. Sensitivity analyses showed no difference between maternal and paternal grandparents’ social influence on offspring’s ARDs.

To assess the effect of intergenerational social mobility, five general trajectories of grandparental-to-parental social classes were constructed: (i) “stable highly advantaged” if both generations belonged to highly advantaged; (ii) “downwardly mobile” if grandparental-to-parental changes were from advantaged to disadvantaged or from highly advantaged down to advantage or disadvantaged; (iii) “upwardly mobile” if transitioned from advantaged to highly advantaged or from disadvantaged up to advantaged or highly advantaged; (iv) “stable advantaged” if grandparents and parents belonged to advantaged; and (v) “stable disadvantaged” if both generations belonged to disadvantaged category. Additionally, we analysed upward trajectories with “highly advantaged” social class as a destination point.

### Outcomes

To form the set of ARDs, the “alcohol index” (i.e. the list of diagnoses used for reporting official statistics on prevalence and trends in alcohol-related hospitalization and mortality in Sweden) introduced by the National Board of Health and Welfare [46] was used, but restricted to diagnoses corresponding to the effect of long-term alcohol misuse. Diagnoses were identified by the codes from the International Classification of Diseases (ICD) (ICD-10: E24.4, F10.1–10.9, G31.2, G62.1, G72.1, I42.6, K29.2, K70, K85.2, K86.0, O35.4, T51, Z50.2, Z71.4, Z72.1 and the corresponding codes from the ICD-9^th^, 8^th^ and 7^th^ revisions) and further subdivided into two groups—mental and behavioural ARDs and other ARDs. Details on classification are presented in Table 2 footnotes. ARDs in offspring were indicated by the first entry in the National Patient Register on alcohol-related main or supplementary diagnoses from inpatient care (1964–2008) and outpatient care (1997–2008) and by the entry in the National Cause of Death register (1964–2008) on alcohol-related main or contributory death cause.

**Table 2:** Incident cases of alcohol-related disorders (ARD) in offspring in population I (G2) and population II (G3) stratified by gender: The Uppsala Birth Cohort Multigenerational Study (UBCoS Multigen).

	Population I (G2)				Population II (G3)			
	Males (n = 9420)		Females (n = 9010)		Males (n = 13 575)		Females (n = 12 894)	
	Cases	Crude incidence rates per 10 000 person-years	Cases	Crude incidence rates per 10 000 person-years	Cases	Crude incidence rates per 10 000 person-years	Cases	Crude incidence rates per 10 000 person-years
**Total incident ARD cases**	544	14.46 (13.30, 15.73)	249	6.83 (6.03, 7.74)	227	9.42 (8.27, 10.72)	176	7.69 (6.63, 8.91)
**Mental and behavioural ARDs**^a^	478	12.71 (11.62, 13.90)	204	5.60 (4.88, 6.42)	150	6.22 (5.30, 7.30)	102	4.56 (3.67, 5.41)
**Other ARDs**^b^	66	1.76 (1.38, 2.23)	45	1.23 (0.92, 1.65)	77	3.19 (2.55, 3.99)	74	3.23 (2.57, 4.06)
**ARDs in age groups**								
12–19	21	3.76 (2.45, 5,77)	21	3.97 (2.59, 6.08)	60	5.56 (4.32, 7.16)	74	7.24 (5.76, 9.09)
20–29	109	11.91 (9.87, 14.36)	31	3.54 (2.49, 5.04)	105	10.94 (9.03, 13.24)	64	7.04 (5.51, 8.99)
30–39	119	13.31 (11.12, 15.93)	54	6.26 (4.79, 8.17)	56	15.94 (12.27, 20.71)	32	9.47 (6.70, 13.39)
40–49	131	15.84 (13.34, 18.79)	72	8.90 (7.06, 11.21)	6	28.54 (12.82, 63.53)	6	30.02 (13.49, 66.83)
50–59	139	28.35 (24.01, 33.48)	62	12.75 (9.94, 16.36)	---	---	---	---
60 +	25	33.02 (22.31, 48.86)	9	11.02 (5.73, 21.18)	---	---	---	---

^a^ Mental and behavioural ARDs’ ICD codes include F10.1–10.9 (ICD-10) and the corresponding codes from the ICD-9^th^, 8^th^ and 7^th^ revisions. Most common mental and behaviour ARDs in the G2 males and females: alcohol dependence (74.5% and 71.6%, respectively), harmful use (16.3% and 18.1%) and withdrawal state (3.6% and 3.4%); in the G3 males and females: harmful use (50.0% and 47.1%), alcohol dependence (36.0% and 41.2%) and unspecified mental and behaviour disorders due to alcohol (8.7% and 5.9%).

^b^ Other ARDs’ ICD codes include E24.4, G31.2, G62.1, G72.1, I42.6, K29.2, K70, K85.2, K86.0, O35.4, T51, Z50.2, Z71.4, Z72.1 (ICD-10) and the corresponding codes from the ICD-9^th^, 8^th^ and 7^th^ revisions. Most common other ARDs in the G2 males and females: toxic effect of ethanol (45.5% and 55.6%, respectively), toxic effect of unspecified alcohol (16.7% and 13.3%) and alcoholic cirrhosis of liver (6.0% and 8.8%); in the G3 males and females: toxic effect of unspecified alcohol (35.1% and 55.4%), toxic effect of ethanol (54.5% and 33.8%) and problems related to life-style due to alcohol use (3.9% and 4.1%).

### Intergenerational covariates

Grandmother’s and mother’s marital status was established from the archived hospital obstetric records for the G0s and from the Population and Housing Census 1960–1990 for the G1s and G2s near the time they gave birth. The G0’s marital status was defined as “married” and “unmarried”, while for the G1-G2s as “married or cohabiting” and “other”. In populations I and II parental educational attainment of the G1s and G2s was retrieved from the Population and Housing Census 1960–1990 and the Longitudinal Integration Database for Medical Insurance and Labour Market Studies (LISA) (1990–2010) [47] for the age of 21 and above and categorised as “tertiary”, “secondary”, “elementary or none”. It was then combined within a couple towards the parent with the highest education to generate a single measure for each offspring. The same registers were used to obtain average age- and sex-standardised disposable income, equivalised for family composition, among mothers and fathers during their working life (age 25–64). Income was then averaged over two parents to acquire a household measure and converted into quartiles of mean household income. Maternal and paternal ARD history was indicated by at least one lifetime entry in the National Patient Register and the National Cause of Death register on ARDs defined by the same ICD codes as indicated for offspring. Offspring birth years were split by 5-year periods.

### Statistical analysis

Cox proportional hazards models were fitted to estimate the hazard ratio (HR) and 95% confidence intervals (CI) for the offspring’s ARDs in relation to grandparental and parental social class. The proportional hazard assumption was checked by the log-rank test for equality of survival function and the log-survival plots and was generally met. The small number of violations was addressed by re-running the analyses using Poisson regression with robust standard errors. The results from the Poisson regression revealed high similarity with the results obtained in the Cox proportional regression analyses, and so we focus on the Cox regression results in this paper.

The National Patient Register was established in 1964, the year that defined the start of follow-up. As the study aimed at assessing ARDs stemming from long-term alcohol misuse, 12 years was set as a lower age boundary for inclusion. Therefore, person-time (population I: 740 471 person-years; population II: 470 035 person-years) was calculated from January 1, 1964 or from the offspring’s 12^th^ birthday, whichever occurred later, until the date of the first ARD diagnosis, date of death from other causes, date of emigration or until the end of follow-up on the December 31, 2008, whichever occurred first. Tests for interaction indicated that offspring’s gender modified the effect of grandparental and parental social class on the outcome (population I: p-value for heterogeneity <0.001; population II: p = 0.04); therefore, all analyses were performed separately for males and females. To account for non-independence between siblings, the robust standard errors were calculated in the Cox regression models with clustering by study subject’s mother.

The HRs minimally-adjusted for offspring’s birth year were computed to assess the contribution of each predictor and covariate to developing ARDs in offspring. The role of grandparental social class in offspring’s ARDs was determined after additionally controlling for grandmother’s marital status (Model 1). The impact of parental social class on offspring’s ARDs was assessed by adjusting for mother’s marital status along with mother’s and father’s ARDs (Model 2). Finally, we fitted a model adjusted for all above mentioned covariates (Model 3). The effect of grandparent-to-parent social trajectories on offspring’s ARDs was assessed, first, by minimally-adjusting for the offspring’s birth year, and then by additionally controlling for grandmother’s and mother’s marital status and parental ARDs.

In population I and II, parental education and household income were strongly correlated with parental social class (population I: r_edu_ = 0.46 and r_inc_ = 0.40; population II: r_edu_ = 0.48 and r_inc_ = 0.41). Because of these strong correlations, it was not appropriate to model the main and the mediating effects of parental social class, education, and income simultaneously. Instead, we performed sensitivity analyses with education and income as alternative parental social indicators. Additionally, the potential mediating role of parental variables was assessed by seemingly unrelated regression and bootstrapping [48] to estimate the proportions of total effect of grandparental social class on offspring’s ARDs mediated by parental social class, education and income. Furthermore, we performed an additional sensitivity analysis by restricting the follow-up time for population I to offspring’s 44^th^ birthday and re-running abovementioned models. We did this because 43 years was the maximum age reached by subjects in population II. Finally, we checked the robustness of our results by additionally adjusting the models for grandparents’ own ARDs. This analysis was conducted only for population II as grandparental diagnoses for population I were not available. All analyses were performed by using STATA 13.1 (Stata Corp, College Station, TX, USA). The STROBE checklist is presented in S1 File.

## Results

### Incidence of ARDs in offspring

In total, 793 incident ARD cases were identified in the G2 subjects (544 among males) and 403 cases among the G3 subjects (227 among males). Table 2 shows the distribution of cases along with incidence rates estimated separately for males and females and subdivided by type of the diagnoses and age at diagnoses. As anticipated, in both populations, incidence rates in males were higher than those in females, although this was not true in the group diagnosed at the youngest age (12–19 years) of the G2s and G3s and at the age of 40–49 years of the G3s. Interestingly, the gender difference in rates was narrower among the G3s than the G2s. In both populations, mental and behaviour disorders were the most common type of diagnosis recorded. Distribution of offspring’s ARDs by grandparental and parental study characteristics are presented in S1 and S2 Tables.

### Grandparental and parental social class and ARD incidence in offspring

In population I, disadvantaged social class in grandparents predicted the incidence of ARDs in the G2s, though the association remained consistent through all adjustment models only in females (Table 3). In the G2 males, adjustment for parental social class and other parental covariates attenuated the effect of social class of grandparents. Disadvantaged social class of parents was associated with incidence of ARDs in the G2 males, but not in females. Mother’s marital status at birth and paternal and maternal ARD history appeared to be strong predictors of ARDs in both genders.

**Table 3:** Hazard ratios (HR) and 95%CI for alcohol-related disorders (ARD) in offspring in population I (G2) by grandparental (G0) and parental (G1) social classes stratified by gender: The Uppsala Birth Cohort Multigenerational Study (UBCoS Multigen).

	Population I (G2) Males (n = 9420)				Population I (G2) Females (n = 9010)			
	HR (95% CI)				HR (95% CI)			
	Min adjusted^a^	Model 1^b^	Model 2^b^	Model 3^b^	Min adjusted^a^	Model 1^b^	Model 2^b^	Model 3^b^
**Grandparental social class**								
Highly advant.	1.00***	1.00***		1.00**	1.00**	1.00**		1.00**
Advantaged	1.07 (0.73, 1.56)	1.07 (0.73, 1.56)		0.91 (0.62, 1.35)	1.00 (0.57, 1.78)	1.01 (0.57, 1.78)		1.09 (0.61, 1.94)
Disadvantaged	1.61 (1.14, 2.28)	1.60 (1.13, 2.27)		1.32 (0.93, 1.89)	1.69 (1.02, 2.81)	1.73 (1.04, 2.88)		1.80 (1.07, 3.03)
**Grandmother’s marital status**								
Married	1.00	1.00		1.00	1.00	1.00		1.00
Unmarried	1.17 (0.94, 1.45)	1.04 (0.83, 1.29)		0.99 (0.80, 1.24)	1.06 (0.78, 1.45)	0.91 (0.66, 1.25)		0.88 (0.64, 1.21)
**Parental social class**								
Highly advant.	1.00***		1.00***	1.00**	1.00		1.00	1.00
Advantaged	1.35 (1.01, 1.81)		1.32 (0.99, 1.76)	1.30 (0.97, 1.73)	1.01 (0.65, 1.56)		0.99 (0.64, 1.53)	0.95 (0.61, 1.47)
Disadvantaged	1.44 (1.19, 1.75)		1.47 (1.21, 1.78)	1.42 (1.17, 1.73)	0.94 (0.72, 1.23)		0.99 (0.76, 1.29)	0.94 (0.72, 1.23)
**Mother’s marital status**								
Married/cohab.	1.00**		1.00*	1.00*	1.00***		1.00***	1.00***
Other	1.55 (1.19, 2.03)		1.39 (1.05, 1.82)	1.36 (1.04, 1.79)	2.52 (1.76, 3.61)		2.18 (1.52, 3.12)	2.18 (1.52, 3.12)
**Father’s ARD**								
Never	1.00***		1.00***	1.00***	1.00***		1.00***	1.00***
Ever	2.78 (2.17, 3.56)		2.49 (1.94, 3.21)	2.41 (1.87, 3.11)	2.63 (1.89, 3.66)		2.16 (1.56, 3.00)	2.09 (1.50, 2.91)
**Mother’s ARD**								
Never	1.00***		1.00***	1.00***	1.00***		1.00***	1.00***
Ever	3.27 (2.17, 4.91)		2.55 (1.70, 3.83)	2.59 (1.74, 3.84)	3.85 (2.37, 6.25)		2.97 (1.86, 4.72)	3.03 (1.90, 4.83)

^a^ Adjusted for the birth year of the G2.

^b^ Models 1–3 adjusted for the birth year of the G2 and mutually adjusted for all variables in the column.

(*)p<0.10,

*p<0.05,

**p<0.01,

***p<0.001 in tests for heterogeneity (between the Hazard ratios corresponding to different categories of each explanatory variable).

In population II, no effect of grandparental social class was detected on the G3’s ARDs regardless of gender (Table 4). As in population I, disadvantaged parental social class increased the risk of ARDs in male offspring, but not in females. Grandmother’s and mother’s marital status were consistently associated with incident ARDs only in the G3 females. Maternal history of ARDs remained a strong predictor of alcohol-related problems in all offspring, but the effect of father’s ARD history was evident only in the G3 males.

**Table 4:** Hazard ratios (HR) and 95%CI for alcohol-related disorders (ARD) in offspring in population II (G3) by grandparental (G1) and parental (G2) social classes stratified by gender: The Uppsala Birth Cohort Multigenerational Study (UBCoS Multigen).

	Population II (G3) Males (n = 13 575)				Population II (G3) Females (n = 12 894)			
	HR (95% CI)				HR (95% CI)			
	Min adjusted^a^	Model 1^b^	Model 2^b^	Model 3^b^	Min adjusted^a^	Model 1^b^	Model 2^b^	Model 3^b^
**Grandparental social class**								
Highly advant.	1.00	1.00		1.00	1.00	1.00(*)		1.00
Advantaged	1.28 (0.84, 1.96)	1.27 (0.83, 1.95)		1.15 (0.75, 1.77)	0.68 (0.38, 1.23)	0.67 (0.37, 1.20)		0.64 (0.35, 1.16)
Disadvantaged	1.30 (0.97, 1.72)	1.33 (1.00, 1.76)		1.16 (0.87, 1.55)	1.22 (0.89, 1.66)	1.26 (0.92, 1.72)		1.18 (0.85, 1.65)
**Grandmother’s marital status**								
Married	1.00(*)	1.00*		1.00	1.00*	1.00*		1.00*
Unmarried	1.47 (0.98, 2.23)	1.52 (1.01, 2.29)		1.35 (0.90, 2.03)	1.60 (1.03, 2.50)	1.72 (1.10, 2.69)		1.60 (1.02, 2.50)
**Parental social class**								
Highly advant.	1.00***		1.00**	1.00*	1.00*		1.00*	1.00(*)
Advantaged	1.60 (1.07, 2.38)		1.52 (1.02, 2.27)	1.48 (0.99, 2.21)	0.89 (0.59, 1.33)		0.86 (0.57, 1.29)	0.83 (0.55, 1.26)
Disadvantaged	2.22 (1.49, 3.30)		1.90 (1.26, 2.86)	1.80 (1.19, 2.72)	1.44 (0.97, 2.12)		1.31 (0.88, 1.95)	1.24 (0.82, 1.89)
**Mother’s marital status**								
Married/cohab.	1.00*		1.00	1.00	1.00**		1.00*	1.00*
Other	1.44 (1.09, 1.91)		1.24 (0.94, 1.65)	1.22 (0.92, 1.62)	1.59 (1.13, 2.25)		1.48 (1.04, 2.10)	1.46 (1.03, 2.07)
**Father’s ARD**								
Never	1.00***		1.00***	1.00***	1.00		1.00	1.00
Ever	2.97 (2.10, 4.20)		2.49 (1.73, 3.60)	2.48 (1.72, 3.57)	1.52 (0.89, 2.59)		1.23 (0.70, 2.18)	1.25 (0.71, 2.20)
**Mother’s ARD**								
Never	1.00***		1.00**	1.00**	1.00**		1.00*	1.00*
Ever	2.93 (1.80, 4.76)		2.27 (1.35, 3.81)	2.27 (1.35, 3.81)	2.45 (1.29, 4.62)		2.03 (1.03, 3.99)	1.96 (1.01, 3.81)

^a^ Adjusted for the birth year of the G3.

^b^ Models1-3 adjusted for the birth year of the G3 and mutually adjusted for all variables in the column.

(*)p<0.10,

*p<0.05,

**p<0.01,

***p<0.001 in tests for heterogeneity (between the Hazard ratios corresponding to different categories of each explanatory variable).

### Grandparental-to-parental social trajectories and ARDs in offspring

The analyses of grandparent-to-parent social trajectories indicated that having a family history of stable disadvantaged social circumstances increased the risk for the offspring to develop ARDs when compared to most favourable trajectory (Table 5; general trajectories). In both populations the persistence of social deprivation in association with ARDs was more consistent for males. For the G3 females the association disappeared in the fully-adjusted model. No associations were ever detected for the G2 females. Interestingly, among the upwardly mobile trajectories, no difference in offspring’s ARDs appeared between “stable highly advantaged” category and persons whose ancestors transitioned from advantaged and disadvantaged social classes up to highly advantaged social group (Table 5; upward trajectories).

**Table 5:** Hazard ratios (HR) and 95% CI for alcohol-related disorders in offspring in population I (G2) and population II (G3) by trajectories between grandparental and parental social classes stratified by gender: The Uppsala Birth Cohort Multigenerational Study (UBCoS Multigen).

Trajectories between grandparental and parental social classes	Population I (G2)^a^		Population II (G3)^b^	
	Males (n = 9420)	Females (n = 9010)	Males (n = 13 575)	Females (n = 12 894)
	HR (95% CI)	HR (95% CI)	HR (95% CI)	HR (95% CI)
**General trajectories**				
***Min adjusted***^c^				
Stable highly advantaged	1.00***	1.00*	1.00 (*)	1.00(*)
Downwardly mobile	1.02 (0.65, 1.60)	0.69 (0.35, 1.38)	1.52 (0.95, 2.44)	1.38 (0.82, 2.31)
Upwardly mobile	1.28 (0.86, 1.89)	1.38 (0.80, 2.39)	1.34 (0.82, 2.19)	1.36 (0.80, 2.31)
Stable advantaged	1.13 (0.60, 2.13)	2.11 (0.90, 4.98)	1.42 (0.69, 2.91)	0.47 (0.14, 1.58)
Stable disadvantaged	1.89 (1.27, 2.80)	1.50 (0.86, 2.62)	1.94 (1.19, 3.17)	1.78 (1.04, 3.07)
***Fully adjusted***^d^				
Stable highly advantaged	1.00***	1.00*	1.00	1.00
Downwardly mobile	1.03 (0.66, 1.63)	0.74 (0.37, 1.48)	1.36 (0.84, 2.19)	1.26 (0.74, 2.12)
Upwardly mobile	1.21 (0.82, 1.81)	1.40 (0.80, 2.43)	1.31 (0.80, 2.14)	1.34 (0.79, 2.27)
Stable advantaged	1.12 (0.59, 2.13)	2.27 (0.97, 5.30)	1.34 (0.65, 2.74)	0.44 (0.13, 1.48)
Stable disadvantaged	1.82 (1.22, 2.72)	1.61 (0.90, 2.86)	1.68 (1.02, 2.76)	1.65 (0.96, 2.85)
**Upward trajectories (all trajectories end with “highly advantaged”)**				
***Min adjusted***^c^				
Stable highly advantaged	1.00	1.00**	1.00	1.00
Advantaged to highly advantaged	1.08 (0.66, 1.75)	0.82 (0.41, 1.65)	0.59 (0.14, 2.52)	1.21 (0.41, 3.59)
Disadvantaged to highly advantaged	1.19 (0.78, 1.80)	1.67 (0.96, 2.92)	0.80 (0.35, 1.80)	1.76 (0.88, 3.50)
***Fully adjusted***^d^				
Stable highly advantaged	1.00	1.00*	1.00	1.00
Advantaged to highly advantaged	1.04 (0.64, 1.69)	0.83 (0.41, 1.67)	0.58 (0.14, 2.47)	1.16 (0.38, 3.54)
Disadvantaged to highly advantaged	1.11 (0.71, 1.72)	1.60 (0.91, 2.83)	0.85 (0.37, 1.93)	1.73 (0.87, 3.45)

^a^ In the G2 analysis: the grandparental generation (G0), the parental generation (G1).

^b^ In the G3 analysis: the grandparental generation (G1), the parental generation (G2).

^c^ Adjusted for offspring’s year of birth.

^d^ Adjusted for offspring’s year of birth, grandmother’s marital status, mother’s marital status, father’s alcohol-related disorders ever in life, mother’s alcohol-related disorders ever in life.

(*)p<0.10,

*p<0.05,

**p<0.01,

***p<0.001 in tests for heterogeneity (between the Hazard ratios corresponding to different categories of each explanatory variable).

### Sensitivity and mediation analyses

We conducted sensitivity analyses replacing social class first with parental education (S3 and S4 Tables), then with parental income (S5 and S6 Tables). This did not change the results previously seen in the main analyses. As anticipated, using a shortened follow-up for population I resulted in obtaining slightly lower incidence rates (S7 Table). When offspring in population I (G2) were followed-up to their 44^th^ birthday (i.e. to the same age as offspring in population II (G3)), all associations previously observed for the G2’s ARDs and grandparental and parental social class and trajectories remained significant (S8 and S9 Tables). As in the analyses with longer follow-up, grandparental social disadvantage predicted ARDs in the G2 females only, while intergenerational persistence of social deprivation was associated solely with ARDs in the G2 males. An additional adjustment for grandparents’ own ARDs in population II did not alter any results previously seen in Table 4 for associations between disadvantaged grandparental social class and the G3’s ARDs (in males: fully adjusted HR = 1.22 (95%CI 0.91, 1.64), in females: HR = 1.16 (95%CI 0.82, 1.63)). The new adjustment did not alter associations with disadvantaged parental social class either (in males: fully adjusted HR = 1.76 (95%CI 1.16, 2.68); in females: HR = 1.35 (95%CI 0.88, 2.10)).

We conducted mediation analyses, examining the proportion of the effect of grandparental social class on offspring ARD development that was mediated by each parental socio-economic characteristic in turn. In population I, 62% of the total effect of grandparental social class on offspring’s ARDs was mediated by parental education; 25% by parental social class; and 19% by parental income. In population II, 65% of the total effect of grandparental social class on offspring’s ARDs was mediated by parental education; 61% by parental social class; and 35% by parental income.

## Discussion

Our results indicate that the intergenerational social patterning of ARDs appears to be time contextual and gender-specific. Grandparental social disadvantage increases the risk of ARDs among individuals born in mid-20^th^ century, particularly in females; while for individuals born in late 1960s-1980s ARDs developing seems to be independent of grandparental social class. In contrast, unfavourable social circumstances in parents impact the development of ARDs in males, but not in females, regardless of time period. As anticipated, mother’s marital status at the time of child birth and mother’s and father’s ARD history are the most consistent predictors of offspring’s ARDs in both populations, though the associations are also time contextual. Specifically, in population I all three of these parental covariates considerably increase ARD risk in the offspring, while in population II ARDs in the G3 males are not associated with mother’s marital status and ARDs in the G3 females are not impacted by father’s ARD history.

Grandparental-to-parental persistence of social disadvantage increases the risk of ARDs in male offspring in both populations regardless of time context. Importantly, however, if parents reach the highly advantaged social class then offspring’s ARD risk is comparatively low, regardless of grandparental background.

### Comparison to other studies

Our findings that ARD incidence increases with age, is higher in males than females, and ARD cases are predominantly made up of mental and behaviour disorders are all in line with international and Swedish data on alcohol-related morbidity and mortality [3, 37, 40, 49]. Similarly, the narrower gender gap in ARDs incidence seen in population II is in line with Swedish and other European data showing a declining gender differences in drinking patterns and alcohol-related harm [50, 51].

With respect to our primary findings, regarding the intergenerational social patterning of ARD’s, it is difficult to make direct comparisons with existing literature because other studies on social causation of ARDs are primarily based on data acquired for parents-child relationships [13–16] or examine grandparental and parental socioeconomic indicators as variables to control for [26]. In terms of comparable multigenerational research, the closest comparisons are three-generational studies examining how ancestor’s education, occupational status and income predict health indicators and conditions co-occurring with ARDs, including impaired cognitive ability [29, 30], psychiatric disorders and externalizing behaviour [52–54]. The studies found grandparental social disadvantages to be associated with impaired cognitive ability in grandchildren even after controlling for parental characteristics [29, 30], while the impact of grandparental education and grandparental-to-parental educational mobility on offspring psychiatric disorders and externalizing behaviours appeared to vary in strength and direction of associations depending on the outcome and offspring gender [52–54].

Although we cannot fully clarify the underlying mechanisms of time trends in grandparental social gradient in offspring’s ARDs, our results suggest possible time contextual effects. It has been argued by Mare [24] that an increased and equal access to mainstream education and material and social resources in descendent generations may weaken the role of grandparental social disadvantage. As our measurements of social indicators span a substantial part of 20^th^ century, it is possible that the declining influence of grandparental social background on grandchildren’s health reflects the process of increasing economic prosperity and social equality that was particularly notable in Sweden in the second half of the century following the previous period of economic difficulties [55].

In this study, we were not able to explore directly the grandparents-to-grandchildren transfer of non-material resources, nor to control for grandparental drinking patterns. Drawing on Swedish alcohol policy history, however, we may speculate that the grandparents from populations I and II differed in their norms and attitudes towards alcohol use. Specifically, despite popular temperance movements, Sweden experienced a period of high alcohol consumption in the late 19^th^ century-beginning of 20^th^ century [56], i.e. during childhood and adolescence of the G0s (grandparents in population I). By contrast, the G1s (grandparents in population II) grew up after 1920, when following the recently established governmental monopoly on alcohol sales, Sweden introduced a restrictive state policy on alcohol (a ‘ration-book system’) reducing access to strong beverages and thereby reducing heavy drinking among males, in particular those of lower social status [56]. In light of the reported relation between high alcohol consumption in adulthood and the experience of growing up during periods of liberal Swedish alcohol policies [38, 39], a more tolerant attitude to alcohol use among the G0s might be assumed. Given a social gradient in alcohol-attributable harm shown in life-course studies [6, 8, 57], it is possible that greater tolerance was differentially patterned across social groups with disadvantaged G0s specifically having had and transmitted a more tolerant view towards alcohol consumption.

Our evidence of association between parental social status and offspring’s ARDs supports the results of other studies on this issue [13–16], while the gender-specific nature of the gradient substantiates the interaction effect between male gender and parental social deprivation reported by Gauffin [58]. The fact that significant associations between social disadvantage in parents and ARDs were only observed in males may reflect different pathways, through which early life disadvantages affect the health of offspring of different genders. For example, studies of adolescent mental health indicate that childhood disadvantage predicts developing internalizing problems in females and externalizing behaviours in males [59, 60] with the latter being also predictive of alcohol-related problems [17].

Sweden is known for its egalitarian approach to income distribution, welfare policies and access to free education. Therefore, studying the effect of social mobility on health meets the concerns of both public health research and practice as it examines whether combating social and economic inequalities can reduce health inequalities. Given the expanding access to education and the labour market during the 20^th^ century, our results highlight the fact that persistence of unfavorable social circumstances in grandparental and parental generations results in an increased risk of ARDs in male offspring regardless of the time context. While it is unclear whether persons with stable disadvantaged grandparent-to-parent social trajectories constitute a group isolated from mainstream societal benefits or reflect accumulation within-family of detrimental medical, psychological and behavioral determinants, targeting these offspring by preventive actions is clearly of importance. Current lack of evidence on social gradient in effectiveness of alcohol prevention and imprecise targeting are considered as substantial obstacles for reaching individuals at-risk [21, 61], though this knowledge is crucial for choosing preventive strategies. Universal interventions benefit socially disadvantaged areas (e.g. deprived community, schools) [61]; while selective interventions focusing on individual risk profile and accounting for contextual role of social environment offer targeted help to those at highest risk [20].

In this context, it is also important to emphasize the beneficial effect of parental transition to highly advantaged social category irrespective of the grandparental social class. This finding resonates with the key points of a recent review, which proposes a forward-thinking three-generation approach to breaking the cycle of intergenerational transmission of disadvantages [19]. This novel approach incorporates measures for interrupting intergenerational chain of poverty in the current generations of parents and children with clinical, policy-oriented and research investments in health of future generation [19].

### Strengths and limitations

The UBCoS Multigen represents a unique set of prospectively collected archive and register-based medical and social records, obtained for representative sample of Swedish individuals and their descendants. The study design limits the potential for information and selection bias, and a large sample size allowed us to perform the analyses in two separate populations stratified by gender. Furthermore, we believe that outcome data collected by the means of the National Patient Register (inpatient and outpatient care, main and supplementary diagnoses) and retrieved from the Cause of Death Register (main and contributory death causes) ensured the inclusion of cases with different levels of severity corresponding to long-term alcohol use. We should, however, acknowledge that after being founded in 1964, the National Patient Register did not reach 100% coverage until 1987, and that psychiatric diagnoses were recorded starting from 1973 [62]. We therefore cannot rule out the risk of missing potential cases occurring before 1987, and also recognize that our collection of the ICD codes from 1964–1973 may underrepresent mental and behavior ARDs.

There are some other methodological limitations. We are unable to rule out potential differential misclassification if persons from different social classes or from different historical periods varied systematically in how likely they were to seek medical help for ARDs, or to receive an ARD diagnosis. However, as we were interested in the effect of long-term alcohol use, and thus collected data on relatively severe ARD cases, we doubt that a large number of people affected by ARD abstained from medical treatment. We also lack data on grandparental ARDs (in population I) and drinking behaviors (in both populations), and are therefore unable to fully examine how far these factors confounded or mediated the observed associations with offspring ARD. Nevertheless, in population II the social patterning of offspring’s ARDs appeared to remain unchanged when grandparental history of ARDs was additionally controlled for. This issue, however, deserves further attention due to the potential complexity of associations between social disadvantage and alcohol-related harm, including the potential for social disadvantage to predispose the development of ARDs (the ‘stress’ hypothesis) [7, 10, 63], the potential for ARDs to cause downward social mobility (the ‘drift’ hypothesis) [64–66], and the potential for any association to be confounded by individual and familial factors [64, 67]. We should also acknowledge that stable social disadvantages might be driven by various unmeasured forces beyond socio-economic determinants that could also confound the associations under the study.

## Conclusions

Exploring social causation of health inequalities requires identification of factors that may facilitate or disrupt the persistence of unfavourable social circumstances across generations. Our study highlights the importance of intergenerational, time contextual and gender-specific perspectives in understanding the social patterning of ARDs. The role of grandparental social class in developing ARDs in grandchildren seems to decline over time, while lower parental social class and persistent grandparental-to-parental social disadvantage remains associated with higher ARD risk in male offspring. It is important to remember that ARDs may, in turn, negatively impact an individual’s own social attainment thereby potentially creating a vicious cycle between social deprivation and alcohol-related harm transmitted from one generation to another. Therefore, when targeting the groups at higher risk of developing ARDs, continuity of familial social disadvantage, particularly among males, should be considered.

## Supporting information

S1 Table Incidence rates, hazard ratios (HR) and 95% CI for alcohol-related disorders (ARD) in offspring in population I (G2) by grandparental (G0) and parental (G1) study characteristics stratified by gender. (DOCX)

S2 Table Incidence rates, hazard ratios (HR) and 95% CI for alcohol-related disorders (ARD) in offspring in population II (G3) by grandparental (G1) and parental (G2) study characteristics stratified by gender. (DOCX)

S3 Table Hazard ratios (HR) and 95%CI for alcohol-related disorders (ARD) in offspring in population I (G2) by grandparental (G0) social classes and parental (G1) education stratified by gender. (DOCX)

S4 Table Hazard ratios (HR) and 95%CI for alcohol-related disorders (ARD) in offspring in population II (G3) by grandparental (G1) and parental (G2) education stratified by gender. (DOCX)

S5 Table Hazard ratios (HR) and 95%CI for alcohol-related disorders (ARD) in offspring in population I (G2) by grandparental (G0) social classes and parental (G1) income stratified by gender. (DOCX)

S6 Table Hazard ratios (HR) and 95%CI for alcohol-related disorders (ARD) in offspring in population II (G3) by grandparental (G1) and parental (G2) income stratified by gender. (DOCX)

S7 Table Incident cases of alcohol-related disorders (ARD) in offspring in population I (G2) occurring up to the age of 44 years and population II (G3) stratified by gender. (DOCX)

S8 Table Hazard ratios (HR) and 95%CI for alcohol-related disorders (ARD) in offspring in population I (G2) up to the age of 44 years by grandparental (G0) and parental (G1) social classes stratified by gender. (DOCX)

S9 Table Hazard ratios (HR) and 95% CI for alcohol-related disorders (ARD) in offspring in population I (G2) up to the age of 44 years by trajectories between grandparental and parental social classes stratified by gender. (DOCX)

S1 File STROBE Statement—Checklist of items that should be included in reports of cohort studies. (DOCX)
