# Supplementary material for: Social class, social mobility and alcohol-related disorders in Swedish men and women: A study of four generations
Source: PLoS One. 2018 Feb 14;13(2):e0191855. doi: 10.1371/journal.pone.0191855 (PMC5812607; doi:10.1371/journal.pone.0191855)
Supplement: S1 Table — (DOCX) [file pone.0191855.s001.docx]

**S1 Table. Incidence rates, hazard ratios (HR) and 95% CI for alcohol-related disorders (ARD) in offspring in population I (G2) by grandparental (G0) and parental (G1) study characteristics stratified by gender: the Uppsala Birth Cohort Multigenerational Study (UBCoS Multigen).**

|  | **Population I (G2) Males (n=9420)** | | | | **Population I (G2) Females (n=9010)** | | | |
| --- | --- | --- | --- | --- | --- | --- | --- | --- |
|  | **n** | **Cases** | **Crude incidence rate per 10 000 person-years** | **HR (95% CI)^a^** | **n** | **Cases** | **Crude incidence rate per 10 000 person-years** | **HR (95% CI)^a^** |
| **Offspring birth year** |  |  |  |  |  |  |  |  |
| 1940-1944 | 950 | 62 | 15.69 (12.32, 20.29) | 1.00(*) | 994 | 36 | 8.54 (6.22, 12.03) | 1.00 |
| 1945-1949 | 2510 | 192 | 18.25 (15.82, 21.15) | 1.39 (1.03, 1.89) | 2336 | 72 | 7.19 (5.75, 9.12) | 0.90 (0.60, 1.35) |
| 1950-1954 | 2753 | 172 | 14.93 (12.83, 17.48) | 1.32 (0.96, 1.82) | 2560 | 79 | 7.30 (5.89, 9.15) | 1.03 (0.68, 1.56) |
| 1955-1959 | 2083 | 82 | 10.42 (8.45, 13.01) | 1.06 (0.74, 1.52) | 2048 | 46 | 5.88 (4.44, 7.94) | 0.94 (0.59, 1.49) |
| 1960-1964 | 1124 | 36 | 9.62 (7.02, 13.56) | 1.08 (0.70, 1.68) | 1072 | 16 | 4.49 (2.80, 7.69) | 0.76 (0.41, 1.38) |
| **Grandparental social class^b^** |  |  |  |  |  |  |  |  |
| Highly advantaged | 993 | 38 | 9.83 (7.15, 13.87) | 1.00*** | 901 | 16 | 4.55 (2.85, 7.74) | 1.00** |
| Advantaged | 2502 | 109 | 10.91 (9.00, 13.34) | 1.07 (0.73, 1.56) | 2390 | 45 | 4.68 (3.48, 6.43) | 1.00 (0.57, 1.78) |
| Disadvantaged | 5925 | 397 | 16.72 (15.10, 18.56) | 1.61 (1.14, 2.28) | 5719 | 188 | 8.07 (7.00, 9.35) | 1.69 (1.02, 2.81) |
| **Grandmother’s marital status** |  |  |  |  |  |  |  |  |
| Married | 7596 | 422 | 13.94 (12.63, 15.42) | 1.00 | 7297 | 198 | 6.73 (5.85, 7.78) | 1.00 |
| Unmarried | 1824 | 122 | 16.66 (13.90, 20.13) | 1.17 (0.94, 1.45) | 1713 | 51 | 7.26 (5.55, 9.67) | 1.06 (0.78, 1.45) |
| **Parental social class^c^** |  |  |  |  |  |  |  |  |
| Highly advantaged | 4278 | 198 | 11.65 (10.11, 13.51) | 1.00*** | 4212 | 118 | 6.99 (5.85, 8.42) | 1.00 |
| Advantaged | 1022 | 66 | 16.14 (12.64, 20.94) | 1.35 (1.01, 1.81) | 908 | 27 | 7.22 (4.91, 11.08) | 1.01 (0.65, 1.56) |
| Disadvantaged | 4120 | 280 | 16.9 (15.00, 19.21) | 1.44 (1.19, 1.75) | 3890 | 104 | 6.57 (5.42, 8.05) | 0.94 (0.72, 1.23) |
| **Mother’s marital status** |  |  |  |  |  |  |  |  |
| Married/cohabiting | 8695 | 480 | 13.82 (12.60, 15.20) | 1.00** | 8349 | 207 | 6.13 (5.36, 7.04) | 1.00*** |
| Other | 725 | 64 | 22.23 (17.49, 28.67) | 1.55 (1.19, 2.03) | 661 | 42 | 15.73 (11.45, 22.21) | 2.52 (1.76, 3.61) |
| **Parental education** |  |  |  |  |  |  |  |  |
| Tertiary | 1819 | 65 | 9.26 (7.21, 12.10) | 1.00*** | 1685 | 27 | 4.14 (2.88, 6.16) | 1.00* |
| Secondary | 3267 | 178 | 13.58 (11.71, 15.83) | 1.38 (1.02, 1.86) | 3229 | 101 | 7.73 (6.38, 9.45) | 1.79 (1.17, 2.73) |
| None/Elementary | 4334 | 301 | 17.22 (15.32, 19.42) | 1.70 (1.28, 2.27) | 4096 | 121 | 7.18 (6.00, 8.68) | 1.63 (1.07, 2.49) |
| **Parental income** |  |  |  |  |  |  |  |  |
| 1 (Richest) | 2363 | 116 | 12.25 (10.14, 14.95) | 1.00** | 2304 | 62 | 6.71 (5.27, 8.70) | 1.00 |
| 2 | 2316 | 125 | 13.45 (11.24, 16.23) | 1.11 (0.85, 1.45) | 2294 | 65 | 6.97 (5.48, 9.01) | 1.04 (0.73, 1.47) |
| 3 | 2391 | 133 | 13.94 (11.72, 16.72) | 1.18 (0.91, 1.54) | 2209 | 58 | 6.45 (4.99, 8.48) | 0.97 (0.68, 1.40) |
| 4 (Poorest) | 2350 | 170 | 18.28 (15.67, 21.45) | 1.55 (1.21, 2.00) | 2203 | 64 | 7.20 (5.62, 9.37) | 1.09 (0.76, 1.56) |

**S1 Table (cont.).**

|  | **Population I (G2) Males (n=9420)** | | | | **Population I (G2) Females (n=9010)** | | | |
| --- | --- | --- | --- | --- | --- | --- | --- | --- |
|  | **n** | **Cases** | **Crude incidence rate per 10 000 person-years** | **HR (95% CI)^a^** | **n** | **Cases** | **Crude incidence rate per 10 000 person-years** | **HR (95% CI)^a^** |
| **Father’s ARD** |  |  |  |  |  |  |  |  |
| Never | 8788 | 458 | 13.01 (11.85, 14.32) | 1.00*** | 8352 | 206 | 6.10 (5.32, 7.02) | 1.00*** |
| Ever | 632 | 86 | 35.69 (28.63, 45.01) | 2.78 (2.17, 3.56) | 658 | 43 | 16.19 (12.09, 22.19) | 2.63 (1.89, 3.66) |
| **Mother’s ARD** |  |  |  |  |  |  |  |  |
| Never | 9245 | 516 | 13.97 (12.78, 15.30) | 1.00*** | 8819 | 231 | 6.47 (5.69, 7.39) | 1.00*** |
| Ever | 175 | 28 | 42.33 (28.92, 64.24) | 3.27 (2.17, 4.91) | 191 | 18 | 24.36 (15.59, 40.21) | 3.85 (2.37, 6.25) |
| **General trajectories for G0-G1’s social classes** |  |  |  |  |  |  |  |  |
| Stable highly advantaged | 820 | 31 | 9.79 (6.86, 14.45) | 1.00*** | 736 | 14 | 4.95 (3.00, 8.77) | 1.00* |
| Downwardly mobile | 1318 | 56 | 10.49 (8.10, 13.82) | 1.02 (0.65, 1.60) | 1233 | 18 | 3.57 (2.30, 5.88) | 0.69 (0.35, 1.38) |
| Upwardly mobile | 4118 | 219 | 13.30 (11.61, 15.30) | 1.28 (0.86, 1.89) | 4083 | 120 | 7.24 (6.07, 8.70) | 1.38 (0.80, 2.39) |
| Stable advantaged | 298 | 14 | 11.87 (7.21, 20.94) | 1.13 (0.60, 2.13) | 250 | 11 | 11.02 (5.67, 24.37) | 2.11 (0.90, 4.98) |
| Stable disadvantaged | 2866 | 224 | 19.57 (17.06, 22.54) | 1.89 (1.27, 2.80) | 2708 | 86 | 7.82 (6.33, 9.79) | 1.50 (0.86, 2.62) |
| **Upward trajectories for G0-G1’s social classes (all trajectories end with “highly advantaged”)** |  |  |  |  |  |  |  |  |
| Stable highly advantaged | 820 | 31 | 9.79 (6.86, 14.45) | 1.0 | 736 | 14 | 4.95 (3.00, 8.77) | 1.00** |
| Advantaged to highly advantaged | 1059 | 46 | 11.02 (8.11, 15.35) | 1.08 (0.66, 1.75) | 1072 | 18 | 4.21 (2.70, 6.95) | 0.82 (0.41, 1.65) |
| Disadvantaged to highly advantaged | 2399 | 121 | 12.54 (10.49, 15.11) | 1.19 (0.78, 1.80) | 2404 | 86 | 8.79 (7.14, 10.95) | 1.67 (0.96, 2.92) |

^a^ Adjusted for the G2s’ birth year.

^b^ The G0s’social class was based on male’s occupation, if available; otherwise on female’s occupation and categorised into “highly advantaged” for higher/intermediate nonmanuals, academic professionals; “advantaged” for the self-employed, farmers and lower nonmanuals; and “disadvantaged” for skilled/unskilled manuals. If data on grandparents were available for only one lineage; variable was created with respect to grandparent with the highest social class. If grandparents from both lineages were assessed, social class of one randomly chosen grandparent, i.e. the “available grandparent”, was used for each offspring.

^c^ The G1s’social class was based separately on father’s and mother’s occupation and categorised as “highly advantaged” for higher/intermediate nonmanuals, self-employed in academic professions; “advantage” for the self-employed in industry, trading, transport or service with employees, farmers with employees, lower nonmanuals and persons at the military service; and “disadvantaged” for skilled/unskilled manuals, self-employed in industry, trading, transport or service without employees, farmers without employees, students, persons with unidentified occupation and others. The highest parental social class was defined by comparing maternal and paternal occupations.

(*)p<0.10, *p<0.05, **p<0.01, ***p<0.001 in tests for heterogeneity (between the Hazard ratios corresponding to different categories of each explanatory variable).
