# Supplementary material for: Social class, social mobility and alcohol-related disorders in Swedish men and women: A study of four generations
Source: PLoS One. 2018 Feb 14;13(2):e0191855. doi: 10.1371/journal.pone.0191855 (PMC5812607; doi:10.1371/journal.pone.0191855)
Supplement: S3 Table — (DOCX) [file pone.0191855.s003.docx]

**S3 Table. Hazard ratios (HR) and 95%CI for alcohol-related disorders (ARD) in offspring in population I (G2) by grandparental (G0) social classes and parental (G1) education stratified by gender: the Uppsala Birth Cohort Multigenerational Study (UBCoS Multigen).**

|  | **Population I (G2) Males (n=9420)** | | | | **Population I (G2) Females (n=9010)** | | | |
| --- | --- | --- | --- | --- | --- | --- | --- | --- |
|  | **HR (95% CI)** | | | | **HR (95% CI)** | | | |
|  | **Min adjusted^a^** | **Model 1^b^** | **Model 2^b^** | **Model 3^b^** | **Min adjusted^a^** | **Model 1^b^** | **Model 2^b^** | **Model 3^b^** |
| **Grandparental social class** |  |  |  |  |  |  |  |  |
| Highly advant. | 1.00*** | 1.00*** |  | 1.00** | 1.00** | 1.00** |  | 1.00* |
| Advantaged | 1.07 (0.73, 1.56) | 1.07 (0.73, 1.56) |  | 0.90 (0.60, 1.35) | 1.00 (0.57, 1.78) | 1.01 (0.57, 1.78) |  | 0.95 (0.52, 1.73) |
| Disadvantaged | 1.61 (1.14, 2.28) | 1.60 (1.13, 2.27) |  | 1.28 (0.87, 1.87) | 1.69 (1.02, 2.81) | 1.73 (1.04, 2.88) |  | 1.54 (0.88, 2.67) |
| **Grandmother’s marital status** |  |  |  |  |  |  |  |  |
| Married | 1.00 | 1.00 |  | 1.00 | 1.00 | 1.00 |  | 1.00 |
| Unmarried | 1.17 (0.94, 1.45) | 1.04 (0.83, 1.29) |  | 0.99 (0.80, 1.23) | 1.06 (0.78, 1.45) | 0.91 (0.66, 1.25) |  | 0.88 (0.64, 1.20) |
| **Parental education** |  |  |  |  |  |  |  |  |
| Tertiary | 1.00*** |  | 1.00** | 1.00* | 1.00* |  | 1.00 | 1.00 |
| Secondary | 1.38 (1.02, 1.86) |  | 1.30 (0.96, 1.75) | 1.22 (0.89, 1.68) | 1.79 (1.17, 2.73) |  | 1.64 (1.07, 2.51) | 1.48 (0.94, 2.34) |
| None/Element. | 1.70 (1.28, 2.27) |  | 1.64 (1.23, 2.18) | 1.52 (1.11, 2.07) | 1.63 (1.07, 2.49) |  | 1.54 (1.01, 2.35) | 1.36 (0.86, 2.15) |
| **Mother’s marital status** |  |  |  |  |  |  |  |  |
| Married/cohab. | 1.00** |  | 1.00* | 1.00* | 1.00*** |  | 1.00*** | 1.00*** |
| Other | 1.55 (1.19, 2.03) |  | 1.33 (1.02, 1.75) | 1.32 (1.01, 1.73) | 2.52 (1.76, 3.61) |  | 2.16 (1.51, 3.11) | 2.16 (1.50, 3.11) |
| **Father’s ARD** |  |  |  |  |  |  |  |  |
| Never | 1.00*** |  | 1.00*** | 1.00*** | 1.00*** |  | 1.00*** | 1.00*** |
| Ever | 2.78 (2.17, 3.56) |  | 2.46 (1.91, 3.16) | 2.39 (1.86, 3.08) | 2.63 (1.89, 3.66) |  | 2.12 (1.52, 2.94) | 2.07 (1.49, 2.88) |
| **Mother’s ARD** |  |  |  |  |  |  |  |  |
| Never | 1.00*** |  | 1.00*** | 1.00*** | 1.00*** |  | 1.00*** | 1.00*** |
| Ever | 3.27 (2.17, 4.91) |  | 2.60 (1.73, 3.90) | 2.61 (1.75, 3.89) | 3.85 (2.37, 6.25) |  | 2.89 (1.82, 4.59) | 2.96 (1.86, 4.70) |

^a^ Adjusted for the birth year of the G2.

^b^ Models1-3 adjusted for the birth year of the G2 and mutually adjusted for all variables in the column.

(*)p<0.10, *p<0.05, **p<0.01, ***p<0.001 in tests for heterogeneity (between the Hazard ratios corresponding to different categories of each explanatory variable).
