# Supplementary material for: Social class, social mobility and alcohol-related disorders in Swedish men and women: A study of four generations
Source: PLoS One. 2018 Feb 14;13(2):e0191855. doi: 10.1371/journal.pone.0191855 (PMC5812607; doi:10.1371/journal.pone.0191855)
Supplement: S4 Table — (DOCX) [file pone.0191855.s004.docx]

**S4 Table. Hazard ratios (HR) and 95%CI for alcohol-related disorders (ARD) in offspring in population II (G3) by grandparental (G1) and parental (G2) education stratified by gender: the Uppsala Birth Cohort Multigenerational Study (UBCoS Multigen).**

|  | **Population II (G3) Males (n=13 575)** | | | | **Population II (G3) Females (n=12 894)** | | | |
| --- | --- | --- | --- | --- | --- | --- | --- | --- |
|  | **HR (95% CI)** | | | | **HR (95% CI)** | | | |
|  | **Min adjusted^a^** | **Model 1^b^** | **Model 2^b^** | **Model 3^b^** | **Min adjusted^a^** | **Model 1^b^** | **Model 2^b^** | **Model 3^b^** |
| **Grandparental social class** |  |  |  |  |  |  |  |  |
| Highly advant. | 1.00 | 1.00 |  | 1.00 | 1.00 | 1.00(*) |  | 1.00(*) |
| Advantaged | 1.28 (0.84, 1.96) | 1.27 (0.83, 1.95) |  | 1.13 (0.74, 1.73) | 0.68 (0.38, 1.23) | 0.67 (0.37, 1.20) |  | 0.67 (0.37, 1.21) |
| Disadvantaged | 1.30 (0.97, 1.72) | 1.33 (1.00, 1.76) |  | 1.12 (0.84, 1.49) | 1.22 (0.89, 1.66) | 1.26 (0.92, 1.72) |  | 1.28 (0.92, 1.77) |
| **Grandmother’s marital status** |  |  |  |  |  |  |  |  |
| Married | 1.00(*) | 1.00* |  | 1.00 | 1.00* | 1.00* |  | 1.00* |
| Unmarried | 1.47 (0.98, 2.23) | 1.52 (1.01, 2.29) |  | 1.33 (0.88, 2.00) | 1.60 (1.03, 2.50) | 1.72 (1.10, 2.69) |  | 1.65 (1.06, 2.58) |
| **Parental education** |  |  |  |  |  |  |  |  |
| Tertiary | 1.00*** |  | 1.00** | 1.00*** | 1.00 |  | 1.00 | 1.00 |
| Secondary | 2.02 (1.48, 2.75) |  | 1.87 (1.36, 2.56) | 1.81 (1.31, 2.49) | 0.93 (0.67, 1.28) |  | 0.87 (0.63, 1.20) | 0.82 (0.59, 1.15) |
| None/element. | 2.49 (1.60, 3.88) |  | 2.12 (1.33, 3.36) | 2.03 (1.28, 3.22) | 1.20 (0.69, 2.06) |  | 1.05 (0.60, 1.82) | 0.97 (0.56, 1.69) |
| **Mother’s marital status** |  |  |  |  |  |  |  |  |
| Married/cohab. | 1.00* |  | 1.00 | 1.00 | 1.00** |  | 1.00* | 1.00* |
| Other | 1.44 (1.09, 1.91) |  | 1.22 (0.92, 1.62) | 1.20 (0.90, 1.60) | 1.59 (1.13, 2.25) |  | 1.53 (1.08, 2.18) | 1.51 (1.06, 2.14) |
| **Father’s ARD** |  |  |  |  |  |  |  |  |
| Never | 1.00*** |  | 1.00*** | 1.00*** | 1.00 |  | 1.00 | 1.00 |
| Ever | 2.97 (2.10, 4.20) |  | 2.50 (1.74, 3.61) | 2.48 (1.72, 3.57) | 1.52 (0.89, 2.59) |  | 1.32 (0.74, 2.34) | 1.33 (0.75, 2.34) |
| **Mother’s ARD** |  |  |  |  |  |  |  |  |
| Never | 1.00*** |  | 1.00** | 1.00** | 1.00** |  | 1.00* | 1.00* |
| Ever | 2.93 (1.80, 4.76) |  | 2.23 (1.33, 3.74) | 2.22 (1.32, 3.74) | 2.45 (1.29, 4.62) |  | 2.17 (1.10, 4.26) | 2.10 (1.08, 4.09) |

^a^ Adjusted for the birth year of the G3.

^b^ Models1-3 adjusted for the birth year of the G3 and mutually adjusted for all variables in the column.

(*)p<0.10, *p<0.05, **p<0.01, ***p<0.001 in tests for heterogeneity (between the Hazard ratios corresponding to different categories of each explanatory variable).
