# Supplementary material for: Social class, social mobility and alcohol-related disorders in Swedish men and women: A study of four generations
Source: PLoS One. 2018 Feb 14;13(2):e0191855. doi: 10.1371/journal.pone.0191855 (PMC5812607; doi:10.1371/journal.pone.0191855)
Supplement: S5 Table — (DOCX) [file pone.0191855.s005.docx]

**S5 Table. Hazard ratios (HR) and 95%CI for alcohol-related disorders (ARD) in offspring in population I (G2) by grandparental (G0) social classes and parental (G1) income stratified by gender: the Uppsala Birth Cohort Multigenerational Study (UBCoS Multigen).**

|  | **Population I (G2) Males (n=9420)** | | | | **Population I (G2) Females (n=9010)** | | | |
| --- | --- | --- | --- | --- | --- | --- | --- | --- |
|  | **HR (95% CI)** | | | | **HR (95% CI)** | | | |
|  | **Min adjusted^a^** | **Model 1^b^** | **Model 2^b^** | **Model 3^b^** | **Min adjusted^a^** | **Model 1^b^** | **Model 2^b^** | **Model 3^b^** |
| **Grandparental social class** |  |  |  |  |  |  |  |  |
| Highly advant. | 1.00*** | 1.00*** |  | 1.00** | 1.00** | 1.00** |  | 1.00** |
| Advantaged | 1.07 (0.73, 1.56) | 1.07 (0.73, 1.56) |  | 0.98 (0.66, 1.46) | 1.00 (0.57, 1.78) | 1.01 (0.57, 1.78) |  | 1.12 (0.62, 2.01) |
| Disadvantaged | 1.61 (1.14, 2.28) | 1.60 (1.13, 2.27) |  | 1.44 (1.00, 2.07) | 1.69 (1.02, 2.81) | 1.73 (1.04, 2.88) |  | 1.87 (1.10, 3.18) |
| **Grandmother’s marital status** |  |  |  |  |  |  |  |  |
| Married | 1.00 | 1.00 |  | 1.00 | 1.00 | 1.00 |  | 1.00 |
| Unmarried | 1.17 (0.94, 1.45) | 1.04 (0.83, 1.29) |  | 0.99 (0.79, 1.23) | 1.06 (0.78, 1.45) | 0.91 (0.66, 1.25) |  | 0.88 (0.65, 1.22) |
| **Parental income** |  |  |  |  |  |  |  |  |
| 1 (Richest) | 1.00** |  | 1.00* | 1.00 | 1.00 |  | 1.00 | 1.00 |
| 2 | 1.11 (0.85, 1.45) |  | 1.10 (0.84, 1.43) | 1.03 (0.79, 1.36) | 1.04 (0.73, 1.47) |  | 0.99 (0.70, 1.41) | 0.91 (0.64, 1.30) |
| 3 | 1.18 (0.91, 1.54) |  | 1.14 (0.88, 1.49) | 1.08 (0.82, 1.42) | 0.97 (0.68, 1.40) |  | 0.91 (0.63, 1.31) | 0.83 (0.57, 1.20) |
| 4 (Poorest) | 1.55 (1.21, 2.00) |  | 1.40 (1.09, 1.80) | 1.33 (1.03, 1.73) | 1.09 (0.76, 1.56) |  | 0.92 (0.64, 1.32) | 0.85 (0.58, 1.23) |
| **Mother’s marital status** |  |  |  |  |  |  |  |  |
| Married/cohab. | 1.00** |  | 1.00(*) | 1.00 | 1.00*** |  | 1.00*** | 1.00*** |
| Other | 1.55 (1.19, 2.03) |  | 1.28 (0.97, 1.68) | 1.27 (0.97, 1.66) | 2.52 (1.76, 3.61) |  | 2.20 (1.54, 3.16) | 2.22 (1.55, 3.19) |
| **Father’s ARD** |  |  |  |  |  |  |  |  |
| Never | 1.00*** |  | 1.00*** | 1.00*** | 1.00*** |  | 1.00*** | 1.00*** |
| Ever | 2.78 (2.17, 3.56) |  | 2.42 (1.88, 3.11) | 2.34 (1.82, 3.01) | 2.63 (1.89, 3.66) |  | 2.17 (1.56, 3.03) | 2.10 (1.51, 2.93) |
| **Mother’s ARD** |  |  |  |  |  |  |  |  |
| Never | 1.00*** |  | 1.00*** | 1.00*** | 1.00*** |  | 1.00*** | 1.00*** |
| Ever | 3.27 (2.17, 4.91) |  | 2.53 (1.69, 3.79) | 2.57 (1.73, 3.82) | 3.85 (2.37, 6.25) |  | 3.00 (1.89, 4.77) | 3.11 (1.96, 4.95) |

^a^ Adjusted for the birth year of the G2.

^b^ Models1-3 adjusted for the birth year of the G2 and mutually adjusted for all variables in the column.

(*)p<0.10, *p<0.05, **p<0.01, ***p<0.001 in tests for heterogeneity (between the Hazard ratios corresponding to different categories of each explanatory variable).
