# Supplementary material for: Social class, social mobility and alcohol-related disorders in Swedish men and women: A study of four generations
Source: PLoS One. 2018 Feb 14;13(2):e0191855. doi: 10.1371/journal.pone.0191855 (PMC5812607; doi:10.1371/journal.pone.0191855)
Supplement: S6 Table — (DOCX) [file pone.0191855.s006.docx]

**S6 Table. Hazard ratios (HR) and 95%CI for alcohol-related disorders (ARD) in offspring in population II (G3) by grandparental (G1) and parental (G2) income stratified by gender: the Uppsala Birth Cohort Multigenerational Study (UBCoS Multigen).**

|  | **Population II (G3) Males (n=13 575)** | | | | **Population II (G3) Females (n=12 894)** | | | |
| --- | --- | --- | --- | --- | --- | --- | --- | --- |
|  | **HR (95% CI)** | | | | **HR (95% CI)** | | | |
|  | **Min adjusted^a^** | **Model 1^b^** | **Model 2^b^** | **Model 3^b^** | **Min adjusted^a^** | **Model 1^b^** | **Model 2^b^** | **Model 3^b^** |
| **Grandparental social class** |  |  |  |  |  |  |  |  |
| Highly advant. | 1.00 | 1.00 |  | 1.00 | 1.00 | 1.00(*) |  | 1.00 |
| Advantaged | 1.28 (0.84, 1.96) | 1.27 (0.83, 1.95) |  | 1.15 (0.75, 1.76) | 0.68 (0.38, 1.23) | 0.67 (0.37, 1.20) |  | 0.64 (0.35, 1.15) |
| Disadvantaged | 1.30 (0.97, 1.72) | 1.33 (1.00, 1.76) |  | 1.17 (0.88, 1.56) | 1.22 (0.89, 1.66) | 1.26 (0.92, 1.72) |  | 1.18 (0.86, 1.62) |
| **Grandmother’s marital status** |  |  |  |  |  |  |  |  |
| Married | 1.00(*) | 1.00* |  | 1.00 | 1.00* | 1.00* |  | 1.00* |
| Unmarried | 1.47 (0.98, 2.23) | 1.52 (1.01, 2.29) |  | 1.35 (0.90, 2.02) | 1.60 (1.03, 2.50) | 1.72 (1.10, 2.69) |  | 1.62 (1.03, 2.52) |
| **Parental income** |  |  |  |  |  |  |  |  |
| 1 (Richest) | 1.00*** |  | 1.00*** | 1.00*** | 1.00 |  | 1.00 | 1.00 |
| 2 | 1.82 (1.16, 2.86) |  | 1.69 (1.08, 2.65) | 1.66 (1.06, 2.60) | 1.30 (0.82, 2.07) |  | 1.24 (0.78, 1.97) | 1.22 (0.77, 1.95) |
| 3 | 2.32 (1.50, 3.59) |  | 2.06 (1.33, 3.18) | 2.00 (1.29, 3.08) | 1.52 (0.96, 2.43) |  | 1.41 (0.89, 2.24) | 1.36 (0.85, 2.18) |
| 4 (Poorest) | 2.85 (1.85, 4.42) |  | 2.50 (1.62, 3.86) | 2.40 (1.56, 3.71) | 1.62 (1.02, 2.59) |  | 1.51 (0.95, 2.39) | 1.45 (0.90, 2.33) |
| **Mother’s marital status** |  |  |  |  |  |  |  |  |
| Married/cohab. | 1.00* |  | 1.00 | 1.00 | 1.00** |  | 1.00* | 1.00* |
| Other | 1.44 (1.09, 1.91) |  | 1.26 (0.95, 1.67) | 1.24 (0.93, 1.64) | 1.59 (1.13, 2.25) |  | 1.48 (1.04, 2.10) | 1.46 (1.03, 2.07) |
| **Father’s ARD** |  |  |  |  |  |  |  |  |
| Never | 1.00*** |  | 1.00*** | 1.00*** | 1.00 |  | 1.00 | 1.00 |
| Ever | 2.97 (2.10, 4.20) |  | 2.42 (1.68, 3.49) | 2.40 (1.66, 3.46) | 1.52 (0.89, 2.59) |  | 1.26 (0.72, 2.22) | 1.26 (0.72, 2.22) |
| **Mother’s ARD** |  |  |  |  |  |  |  |  |
| Never | 1.00*** |  | 1.00** | 1.00** | 1.00** |  | 1.00* | 1.00* |
| Ever | 2.93 (1.80, 4.76) |  | 2.29 (1.38, 3.82) | 2.29 (1.37, 3.81) | 2.45 (1.29, 4.62) |  | 2.09 (1.07, 4.09) | 2.01 (1.04, 3.91) |

^a^ Adjusted for the birth year of the G3.

^b^ Models1-3 adjusted for the birth year of the G3 and mutually adjusted for all variables in the column.

(*)p<0.10, *p<0.05, **p<0.01, ***p<0.001 in tests for heterogeneity (between the Hazard ratios corresponding to different categories of each explanatory variable).
