# Supplementary material for: Social class, social mobility and alcohol-related disorders in Swedish men and women: A study of four generations
Source: PLoS One. 2018 Feb 14;13(2):e0191855. doi: 10.1371/journal.pone.0191855 (PMC5812607; doi:10.1371/journal.pone.0191855)
Supplement: S7 Table — (DOCX) [file pone.0191855.s007.docx]

**S7 Table. Incident cases of alcohol-related disorders (ARD) in offspring in population I (G2) occurring up to the age of 44 years^a^ and population II (G3) stratified by gender: the Uppsala Birth Cohort Multigenerational Study (UBCoS Multigen).**

|  | **Population I (G2)^b^** | | | | **Population II (G3)^c^** | | | |
| --- | --- | --- | --- | --- | --- | --- | --- | --- |
|  | **Males (n=9420)** | | **Females (n=9010)** | | **Males (n=13 575)** | | **Females (n=12 894)** | |
|  | **Cases** | **Crude incidence rates per 10 000 person-years** | **Cases** | **Crude incidence rates per 10 000 person-years** | **Cases** | **Crude incidence rates per 10 000 person-years** | **Cases** | **Crude incidence rates per 10 000 person-years** |
| **Total incident ARD cases** | 308 | 11.34 (10.14, 12.68) | 142 | 5.45 (4.62, 6.42) | 227 | 9.42 (8.27, 10.72) | 176 | 7.69 (6.63, 8.91) |
| **Mental and behavioural ARDs^d^** | 248 | 9.13 (8.06, 10.34) | 98 | 3.76 (3.08, 4.58) | 150 | 6.22 (5.30, 7.30) | 102 | 4.56 (3.67, 5.41) |
| **Other ARDs^e^** | 60 | 2.21 (1.72, 2.85) | 44 | 1.69 (1.26, 2.27) | 77 | 3.19 (2.55, 3.99) | 74 | 3.23 (2.57, 4.06) |
| **ARD in age groups** |  |  |  |  |  |  |  |  |
| 12-19 | 21 | 3.76 (2.45, 5,77) | 21 | 3.97 (2.59, 6.08) | 60 | 5.56 (4.32, 7.16) | 74 | 7.24 (5.76, 9.09) |
| 20-29 | 109 | 11.91 (9.87, 14.36) | 31 | 3.54 (2.49, 5.04) | 105 | 10.94 (9.03, 13.24) | 64 | 7.04 (5.51, 8.99) |
| 30-39 | 119 | 13.31 (11.12, 15.93) | 54 | 6.26 (4.79, 8.17) | 56 | 15.94 (12.27, 20.71) | 32 | 9.47 (6.70, 13.39) |
| 40-43 | 59 | 16.65 (12.88, 21.55) | 36 | 10.61 (7.65, 14.71) | 6 | 28.54 (12.82, 63.53) | 6 | 30.02 (13.49, 66.83) |

^a^ Person-time for sensitivity analysis on population I was calculated from January 1, 1964 or from the offspring’s 12^th^ birthday, whichever occurred later, until the date of the first ARD diagnosis, date of death from other causes, date of emigration or until the offspring’s 44^th^ birthday, whichever occurred first.

^b^ For population I (G2), number of ARD cases and incidence rates are calculated during the follow-up up to the age of 44 years.

^c^ For population II (G3), number of ARD cases and incidence rates are the same as reported in Table 2.

^d^ Mental and behavioural ARDs’ ICD codes include (ICD-10): F10.1-10.9 and the corresponding codes from the ICD-9^th^, 8^th^ and 7^th^ revisions.

^e^ Other ARDs’ ICD codes include (ICD-10): E244, G31.2, G62.1, G72.1, I42.6, K29.2, K70, K85.2, K86.0, O35.4, T51, Z50.2, Z71.4, Z72.1 and the corresponding codes from the ICD-9^th^, 8^th^ and 7^th^ revisions.
