# Supplementary material for: Social class, social mobility and alcohol-related disorders in Swedish men and women: A study of four generations
Source: PLoS One. 2018 Feb 14;13(2):e0191855. doi: 10.1371/journal.pone.0191855 (PMC5812607; doi:10.1371/journal.pone.0191855)
Supplement: S8 Table — (DOCX) [file pone.0191855.s008.docx]

**S8 Table. Hazard ratios (HR) and 95%CI for alcohol-related disorders (ARD) in offspring in population I (G2) up to the age of 44 years^a^ by grandparental (G0) and parental (G1) social classes stratified by gender: the Uppsala Birth Cohort Multigenerational Study (UBCoS Multigen).**

|  | **Population I (G2) Males (n=9420)** | | | | **Population I (G2) Females (n=9010)** | | | |
| --- | --- | --- | --- | --- | --- | --- | --- | --- |
|  | **HR (95% CI)** | | | | **HR (95% CI)** | | | |
|  | **Min adjusted^b^** | **Model 1^c^** | **Model 2^c^** | **Model 3^c^** | **Min adjusted^b^** | **Model 1^c^** | **Model 2^c^** | **Model 3^c^** |
| **Grandparental social class** |  |  |  |  |  |  |  |  |
| Highly advant. | 1.00*** | 1.00** |  | 1.00* | 1.00** | 1.00** |  | 1.00** |
| Advantaged | 1.30 (0.77, 2.20) | 1.29 (0.76, 2.19) |  | 1.01 (0.59, 1.74) | 1.21 (0.52, 2.83) | 1.21 (0.52, 2.83) |  | 1.38 (0.59, 3.23) |
| Disadvantaged | 2.05 (1.27, 3.32) | 1.99 (1.22, 3.24) |  | 1.48 (0.91, 2.43) | 2.45 (1.15, 5.22) | 2.47 (1.16, 5.29) |  | 2.73 (1.27, 5.85) |
| **Grandmother’s marital status** |  |  |  |  |  |  |  |  |
| Married | 1.00 | 1.00 |  | 1.00 | 1.00 | 1.00 |  | 1.00 |
| Unmarried | 1.30 (0.98, 1.72) | 1.13 (0.84, 1.50) |  | 1.05 (0.79, 1.41) | 1.17 (0.78, 1.76) | 0.96 (0.63, 1.45) |  | 0.91 (0.61, 1.37) |
| **Parental social class** |  |  |  |  |  |  |  |  |
| Highly advant. | 1.00*** |  | 1.00*** | 1.00*** | 1.00 |  | 1.00 | 1.00 |
| Advantaged | 1.57 (1.06, 2.30) |  | 1.50 (1.02, 2.21) | 1.46 (0.99, 2.14) | 0.92 (0.50, 1.69) |  | 0.90 (0.49, 1.66) | 0.84 (0.46, 1.53) |
| Disadvantaged | 1.85 (1.43, 2.39) |  | 1.88 (1.45, 2.44) | 1.79 (1.37, 2.33) | 0.91 (0.64, 1.29) |  | 0.98 (0.69, 1.38) | 0.90 (0.63, 1.27) |
| **Mother’s marital status** |  |  |  |  |  |  |  |  |
| Married/cohab. | 1.00** |  | 1.00** | 1.00** | 1.00*** |  | 1.00*** | 1.00*** |
| Other | 1.87 (1.33, 2.63) |  | 1.67 (1.17, 2.37) | 1.63 (1.14, 2.31) | 3.13 (2.03, 4.83) |  | 2.71 (1.76, 4.16) | 2.69 (1.76, 4.12) |
| **Father’s ARD** |  |  |  |  |  |  |  |  |
| Never | 1.00*** |  | 1.00*** | 1.00*** | 1.00*** |  | 1.00** | 1.00* |
| Ever | 3.10 (2.24, 4.30) |  | 2.64 (1.89, 3.68) | 2.54 (1.82, 3.54) | 2.46 (1.56, 3.87) |  | 1.80 (1.17, 2.76) | 1.72 (1.12, 2.64) |
| **Mother’s ARD** |  |  |  |  |  |  |  |  |
| Never | 1.00*** |  | 1.00*** | 1.00*** | 1.00*** |  | 1.00*** | 1.00*** |
| Ever | 3.93 (2.31, 6.66) |  | 2.87 (1.70, 4.88) | 2.91 (1.73, 4.91) | 6.06 (3.59, 10.22) |  | 4.74 (2.90, 7.75) | 4.89 (2.98, 8.01) |

^a^ Person-time for sensitivity analysis on population I was calculated from January 1, 1964 or from the offspring’s 12^th^ birthday, whichever occurred later, until the date of the first ARD diagnosis, date of death from other causes, date of emigration or until the offspring’s 44^th^ birthday, whichever occurred first.

^b^ Adjusted for the birth year of the G2.

^c^ Models 1-3 adjusted for the birth year of the G2 and mutually adjusted for all variables in the column.

(*)p<0.10, *p<0.05, **p<0.01, ***p<0.001 in tests for heterogeneity (between the Hazard ratios corresponding to different categories of each explanatory variable).
