# Supplementary material for: Social class, social mobility and alcohol-related disorders in Swedish men and women: A study of four generations
Source: PLoS One. 2018 Feb 14;13(2):e0191855. doi: 10.1371/journal.pone.0191855 (PMC5812607; doi:10.1371/journal.pone.0191855)
Supplement: S9 Table — (DOCX) [file pone.0191855.s009.docx]

**S9 Table. Hazard ratios (HR) and 95% CI for alcohol-related disorders in offspring in population I (G2) up to the age of 44 years^a^ by trajectories between grandparental and parental social classes stratified by gender: the Uppsala Birth Cohort Multigenerational Study (UBCoS Multigen).**

| **Trajectories between grandparental and parental social classes** | **Population I (G2)^b^** | |
| --- | --- | --- |
|  | **Males (n=9420)** | **Females (n=9010)** |
|  | **HR (95% CI)** | **HR (95% CI)** |
| **General trajectories** |  |  |
| ***Min adjusted^c^*** |  |  |
| Stable highly advantaged | 1.00*** | 1.00* |
| Downwardly mobile | 1.20 (0.64, 2.23) | 0.65 (0.24, 1.78) |
| Upwardly mobile | 1.40 (0.82, 2.40) | 1.76 (0.82, 3.80) |
| Stable advantaged | 1.57 (0.69, 3.55) | 2.05 (0.57, 7.35) |
| Stable disadvantaged | 2.65 (1.55, 4.51) | 1.90 (0.87, 4.17) |
| ***Fully adjusted^d^*** |  |  |
| Stable highly advantaged | 1.00*** | 1.00* |
| Downwardly mobile | 1.22 (0.65, 2.27) | 0.70 (0.26, 1.91) |
| Upwardly mobile | 1.31 (0.76, 2.25) | 1.81 (0.83, 3.93) |
| Stable advantaged | 1.56 (0.69, 3.55) | 2.26 (0.62, 8.19) |
| Stable disadvantaged | 2.50 (1.45, 4.31) | 2.10 (0.94, 4.70) |
| **Upward trajectories (all trajectories end with “highly advantaged”)** |  |  |
| ***Min adjusted^c^*** |  |  |
| Stable highly advantaged | 1.00 | 1.00** |
| Advantaged to highly advantaged | 1.26 (0.65, 2.43) | 0.95 (0.36, 2.47) |
| Disadvantaged to highly advantaged | 1.27 (0.72, 2.26) | 2.15 (0.98, 4.67) |
| ***Fully adjusted^d^*** |  |  |
| Stable highly advantaged | 1.00 | 1.00* |
| Advantaged to highly advantaged | 1.22 (0.63, 1.69) | 0.97 (0.37, 2.57) |
| Disadvantaged to highly advantaged | 1.21 (0.66, 1.72) | 2.05 (0.94, 4.51) |

^a^ Person-time for sensitivity analysis on population I was calculated from January 1, 1964 or from the offspring’s 12th birthday, whichever occurred later, until the date of the first ARD diagnosis, date of death from other causes, date of emigration or until the offspring’s 44th birthday, whichever occurred first.

^b^ In the G2 analysis: the grandparental generation (G0), the parental generation (G1).

^c^ Adjusted for offspring’s year of birth.

^d^ Adjusted for offspring’s year of birth, grandmother’s marital status, mother’s marital status, father’s ARDs ever in life, mother’s ARDs ever in life.

(*)p<0.10, *p<0.05, **p<0.01, ***p<0.001 in tests for heterogeneity (between the Hazard ratios corresponding to different categories of each explanatory variable).
